# CODE-EHR best practice framework for the use of structured electronic healthcare records in clinical research

**DOI:** 10.1093/eurheartj/ehac426

**Published:** 2022-08-29

**Authors:** Dipak Kotecha, Folkert W Asselbergs, Stephan Achenbach, Stefan D Anker, Dan Atar, Colin Baigent, Amitava Banerjee, Birgit Beger, Gunnar Brobert, Barbara Casadei, Cinzia Ceccarelli, Martin R Cowie, Filippo Crea, Maureen Cronin, Spiros Denaxas, Andrea Derix, Donna Fitzsimons, Martin Fredriksson, Chris P Gale, Georgios V Gkoutos, Wim Goettsch, Harry Hemingway, Martin Ingvar, Adrian Jonas, Robert Kazmierski, Susanne Løgstrup, R Thomas Lumbers, Thomas F Lüscher, Paul McGreavy, Ileana L Piña, Lothar Roessig, Carl Steinbeisser, Mats Sundgren, Benoît Tyl, Ghislaine van Thiel, Kees van Bochove, Panos E Vardas, Tiago Villanueva, Marilena Vrana, Wim Weber, Franz Weidinger, Stephan Windecker, Angela Wood, Diederick E Grobbee, the Innovative Medicines Initiative BigData@Heart Consortium, European Society of Cardiology, CODE-EHR international consensus group, Xavier Kurz, John Concato, Robert Kazmierski, Jose Pablo Morales, Ileana Piña, Wim Goettsch, Adrian Jonas, Niklas Hedberg, Filippo Crea, Thomas F Lüscher, Wim Weber, Tiago Villanueva, Stuart Spencer, Rupa Sarkar, Martin Fredriksson, Mats Sundgren, Andrea Derix, Gunnar Brobert, Lothar Roessig, Benoit Tyl, Kees van Bochove, Maureen Cronin, Colm Carroll, Ceri Thompson, Birgit Beger, Susanne Løgstrup, Marilena Vrana, Paul McGreavy, Barbara Casadei, Stephan Achenbach, Valentina Tursini, Panos E Vardas, Dan Atar, Colin Baigent, Chris P Gale, Donna Fitzsimons, Stephan Windecker, Stefan D Anker, Martin Cowie, Amitava Banerjee, Harry Hemingway, R Tom Lumbers, Spiros Denaxas, Folkert W Asselbergs, Rick Grobbee, Ghislaine Van Thiel, Dipak Kotecha, George V Gkoutos, Angela Wood, Martin Ingvar, Carl Steinbeisser, Ana Petrova, Cinzia Ceccarelli, Katija Baljevic, Polyxeni Vairami, Jennifer Taylor

**Affiliations:** Institute of Cardiovascular Sciences, University of Birmingham, Medical School, Birmingham, UK; University Hospitals Birmingham NHS Foundation Trust and Health Data Research UK Midlands, Birmingham, UK; Department of Cardiology, Division of Heart and Lungs, University Medical Centre Utrecht, University of Utrecht, Utrecht, Netherlands; Department of Cardiology, Division of Heart and Lungs, University Medical Centre Utrecht, University of Utrecht, Utrecht, Netherlands; Institute of Cardiovascular Science, Faculty of Population Health Sciences, University College London, London, UK; Health Data Research UK and Institute of Health Informatics, University College London, London, UK; Friedrich-Alexander-Universität Erlangen-Nürnberg (FAU), Erlangen, Germany; Department of Cardiology and Berlin Institute of Health Centre for Regenerative Therapies, German Centre for Cardiovascular Research (DZHK) partner site Berlin; Charité Universitätsmedizin Berlin, Germany; Department of Cardiology, Oslo University Hospital, Ulleval, Oslo, Norway; University of Oslo, Institute of Clinical Medicine, Oslo, Norway; MRC Population Health Research Unit, Nuffield Department of Population Health, Oxford, UK; Clinical Trial Service Unit and Epidemiological Studies Unit, University of Oxford, Oxford, UK; Health Data Research UK and Institute of Health Informatics, University College London, London, UK; University College London Hospitals NHS Trust, London, UK; European Heart Network, Brussels, Belgium; Bayer AB, Stockholm, Sweden; Division of Cardiovascular Medicine, John Radcliffe Hospital, University of Oxford NIHR Oxford Biomedical Research Centre, Oxford, UK; European Society of Cardiology, Sophia Antipolis, France; Royal Brompton Hospital, Division of Guy’s St Thomas’ NHS Foundation Trust, London, UK; School of Cardiovascular Medicine Sciences, King’s College London, London, UK; Department of Cardiovascular and Pulmonary Sciences, Catholic University of the Sacred Heart, Rome, Italy; European Heart Journal, Oxford University Press, University of Oxford, Oxford, UK; Vifor Pharma, Glattbrugg, Switzerland and Ava AG, Zurich, Switzerland; Health Data Research UK and Institute of Health Informatics, University College London, London, UK; Alan Turing Institute, London, UK; British Heart Foundation Data Science Centre, London, UK; Bayer AG, Leverkusen, Germany; School of Nursing and Midwifery, Queen’s University Belfast, Northern Ireland; Late Clinical Development, Cardiovascular, Renal and Metabolism (CVRM), Biopharmaceuticals RD, AstraZeneca, Gothenburg, Sweden; Leeds Institute of Cardiovascular and Metabolic Medicine and Leeds Institute for Data Analytics, University of Leeds, Leeds, UK; Leeds Institute for Data Analytics, University of Leeds, Leeds, UK; Department of Cardiology, Leeds Teaching Hospitals NHS Trust, Leeds, UK; University Hospitals Birmingham NHS Foundation Trust and Health Data Research UK Midlands, Birmingham, UK; College of Medical and Dental Sciences, Institute of Cancer and Genomic Sciences, University of Birmingham, Birmingham, UK; National Health Care Institute (ZIN), Diemen, Netherlands; Division of Pharmacoepidemiology and Clinical Pharmacology, Utrecht Institute for Pharmaceutical Sciences, Utrecht University, Utrecht, Netherlands; Health Data Research UK and Institute of Health Informatics, University College London, London, UK; Department of Clinical Neuroscience, Karolinska Institutet, Solna, Sweden; Department of Neuroradiology, Karolinska University Hospital Stockholm, Stockholm, Sweden; Data and Analytics Group, National Institute for Health and Care Excellence, London, UK; Office of Cardiovascular Devices, US Food and Drug Administration, Silver Spring, MD, USA; European Heart Network, Brussels, Belgium; Health Data Research UK and Institute of Health Informatics, University College London, London, UK; Barts Health NHS Trust and University College London Hospitals NHS Trust; Centre for Molecular Cardiology, University of Zurich, Zurich, Switzerland; Research, Education & Development, Royal Brompton and Harefield Hospitals, London, UK; Faculty of Medicine, Imperial College London, London, UK; European Society of Cardiology Patient Forum, European Society of Cardiology, Brussels, Belgium; Central Michigan University College of Medicine, Midlands, MI, USA; Centre for Devices and Radiological Health, US Food and Drug Administration, Silver Spring, MD, USA; Bayer AG, Leverkusen, Germany; Bayer AG, Leverkusen, Germany; Steinbeisser Project Management, Munich, Germany; Data Science AI, Biopharmaceuticals RD, AstraZeneca, Gothenburg, Sweden; Centre for Therapeutic Innovation, Cardiovascular and Metabolic Disease, Institut de Recherches Internationales Servier, Suresnes, France; Julius Centre for Health Sciences and Primary Care, University Medical Centre Utrecht, Utrecht University, Utrecht, Netherlands; The Hyve, Utrecht, Netherlands; Hygeia, Mitera, Hospitals Hellenic Health Group, Athens, Greece; European Heart Agency, European Society of Cardiology, Brussels, Belgium; The BMJ, London, UK; European Heart Network, Brussels, Belgium; The BMJ, London, UK; Rudolfstiftung Hospital, Vienna, Austria; Department of Cardiology, Inselspital, University Hospital Bern, Bern, Switzerland; Cardiovascular Epidemiology Unit, Department of Public Health and Primary Care, University of Cambridge, Cambridge, UK; Department of Epidemiology, University Medical Centre Utrecht, Division Julius Centrum, Utrecht, Netherlands

## Abstract

Big data is central to new developments in global clinical science aiming to improve the lives of patients. Technological advances have led to the routine use of structured electronic healthcare records with the potential to address key gaps in clinical evidence. The covid-19 pandemic has demonstrated the potential of big data and related analytics, but also important pitfalls. Verification, validation, and data privacy, as well as the social mandate to undertake research are key challenges. The European Society of Cardiology and the BigData@Heart consortium have brought together a range of international stakeholders, including patient representatives, clinicians, scientists, regulators, journal editors and industry. We propose the CODE-EHR Minimum Standards Framework as a means to improve the design of studies, enhance transparency and develop a roadmap towards more robust and effective utilisation of healthcare data for research purposes.

In the context of ageing populations and increasing multimorbidity in all disease areas,^1–3^ large scale, real world data provide an opportunity to better understand the epidemiology of rare and common conditions, and to improve prevention strategies and treatment stratification.^4^ Tailored management for individual patients has become even more essential to constrain healthcare costs and provide patient centred care that can improve a patient's quality of life and prognosis. Embedding controlled trials within the real world setting, either within registries or routine clinical practice, is now possible and could provide more generalisable results to the population at large.^5^

Health data science has undergone rapid development in the past decade, including the common adoption of electronic healthcare record (EHR) systems that condense clinical episodes into a set of coded, structured labels.^6^ However, concerns over quality, data privacy, transparency, and comparability of these systems have limited the use of the evidence generated with structured healthcare data. These issues have also restricted acceptance by regulators, reimbursement authorities, and guideline task forces. Despite the availability of numerous reporting standards, consensus has not been met on how to realise the Findable, Accessible, Interoperable, and Reusable (FAIR) principles^7^ in the context of structured healthcare data. Existing reporting checklists ask authors to indicate where in their paper particular design issues have been discussed. For example, STROBE (Strengthening the Reporting of Observational studies in Epidemiology) for observational studies,^8^ RECORD (REporting of studies Conducted using Observational Routinely-collected Data) for routinely collected health data,^9^ and CONSORT-AI (Consolidated Standards of Reporting Trials-Artificial Intelligence) for artificial intelligence interventions.^10^ However, these checklists are often lengthy, no minimum standards are specified, and adherence does not relate to study quality or even the quality of transparency for that domain.^11^ Although checklists can benefit research quality, they are often used for box ticking to facilitate journal publication. In a study of radiology journals, only 15% (120/821) of surveyed authors used the reporting guideline when designing their study.^12^ With a proliferation of reporting checklists for every scenario, authors and readers of such reports are increasingly confused about the value of these checklists. As of 14 February 2022, 488 reporting checklists were registered with EQUATOR (Enhancing the QUAlity and Transparency Of health Research) and 111 were in development.

In the case of observational and randomised clinical research using EHRs and other structured data, the source of data, its manipulation, and underpinning governance are of critical importance to extrapolating results. Clarity is needed from a broad stakeholder perspective, providing a quality framework to enhance the design and application of clinical research that increasingly depends on these crucial new sources of data. This article reflects the joint work of a wide range of international stakeholders with a remit to improve the use of structured healthcare data. The programme was coordinated by the European Society of Cardiology, a non-profit organisation of healthcare professionals, and the BigData@Heart Consortium, a public-private partnership funded by the European Union Innovative Medicines Initiative. Our aim was to navigate opportunities and limitations, and to develop a framework for a broad audience of global stakeholders across all disease areas. The CODE-EHR framework seeks to realise the exciting opportunity that digitisation of health data affords to increase efficiency of healthcare systems, and improve the lives and wellbeing of patients.

**Summary points**

- Research using routinely collected structured healthcare data has the potential for major clinical impact but this requires a clear and transparent approach to describe data sources, linkage protocols, coding definitions, and validation of methods and results
- A social license and public mandate are essential components of big data research that can provide societal benefit, addressing the concerns of participants, and ensuring data privacy and integrity
- This paper describes the output of international stakeholder meetings for the use of structured healthcare data for research purposes, including patient representatives, clinicians, scientists, regulators, journal editors, and industry representatives
- The CODE-EHR checklist provides a minimum standards framework to enhance research design and enable more effective use and dissemination of routine healthcare data for clinical research

## Stakeholder development of the CODE-EHR framework

A full range of stakeholders participated, including regulators (US Food and Drug Administration, European Medicines Agency), governmental agencies (European Commission, the UK National Institute for Health and Care Excellence, Innovative Medicines Initiative), leading medical journals (*The BMJ*, *European Heart Journal*, *The Lancet*, *The Lancet Digital Health*), and patient advocacy groups (European Heart Network, ESC Patient Forum), in addition to representatives from the pharmaceutical industry, payers, leading academic institutions, and professional societies (see acknowledgments). Development of the CODE-EHR framework was centred on two stakeholder meetings (7 July 2020 and 26 October 2020), consisting of presentations from key opinion leaders, followed by breakout sessions and plenaries to formulate statements on key topic areas. An iterative process with virtual work was used to achieve consensus positions, with a further meeting on 10 March 2022 to finalise this report.

**Figure 1:**
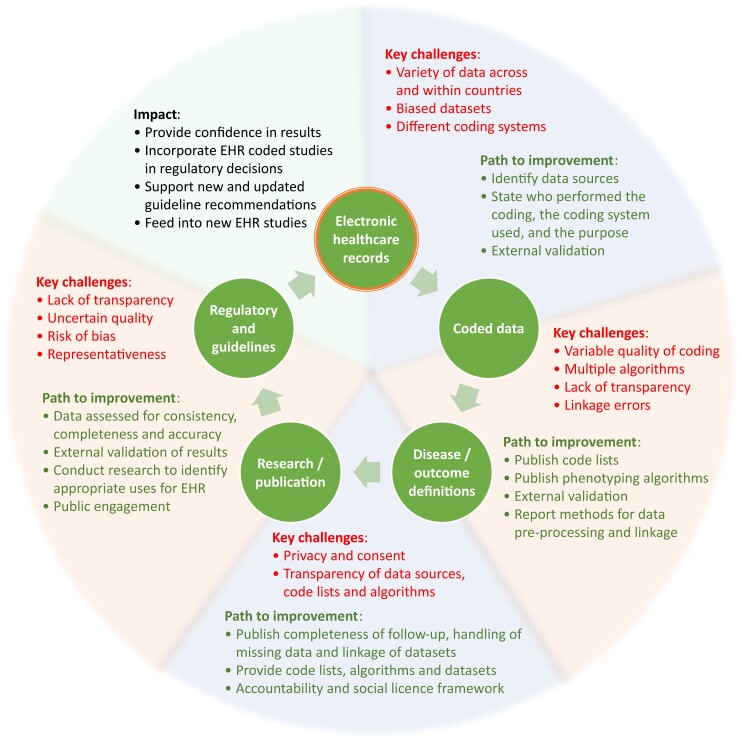
From structured healthcare data to improved patient care. Key challenges and the paths to improvement leading to sustainable impact from EHR-based research studies. EHR=electronic healthcare record.

We aimed to develop pragmatic advice for the use of structured healthcare data within trials and observational studies that is not dependent on particular diseases, and that meets the expectations of stakeholders and the general public. Our objectives were to provide new direction to this increasingly important field in medicine, thereby enhancing the value of routinely collected data to improve future patient wellbeing. Detailed text on the current state-of-the-art for research using healthcare data, in addition to key challenges and limitations, was developed by the stakeholder group, supported by a writing committee. See appendix 1 in which we address the need for common standards to appraise the digital landscape (e.g, coding systems and the vital aspect of linkage), expand on current and future opportunities for the use of structured healthcare data, and show how a social license can lead to co-creation of research with a public health benefit. The key challenges and pathways for improvement are outlined in figure 1, which presents the process of structured healthcare data from initial notation to their potential use to enhance research and subsequently improve clinical practice.

The output of the stakeholder meetings and iterative discussions were condensed into four core central themes: technical process and data stewardship; data security and privacy; publications using structured healthcare data; and addressing the needs of regulators, reimbursement authorities, and clinical practice guidelines. Key statements and advisories from the consensus meetings are summarised in table 1.

**Table 1:** Output from the stakeholder consensus meetings

Workshop theme	Key consensus statements and advisories
1. Technical process and data stewardship	*Research using structured healthcare data conducted according to the FAIR data principles of findability, accessibility, interoperability, and reusability.* ^13^ Important considerations are transparency of who performed the coding, the coding system used, and the purpose of coding (reimbursement, diagnosis, etc.).
	*Clear and consistent identification and description of the sources of EHR data.* Code lists and phenotyping algorithms can be described in detail and published, ideally before a study commences (for example on a coding repository or open-source archive). The minimum data required to meet the definitions will depend on the use case and can be reported to enhance transparency, in addition to the rationale for why certain decisions were made (for example, why one code was chosen over another, or what the effect would be if data collection periods were changed).
	*Validation at local, regional and global levels.* Evidence demonstrating how algorithms have been externally validated, and also what quality assessment was performed on the research findings, for example on the accuracy, completeness and timeliness of the data.^14^ Data quality rules can be used to assess coded data and allow comparisons across institutions and countries.^15^
	*Reporting of methods used for data pre-processing and data linkage.* This includes the methods used to assess the quality of linkage and the results of any data pre-processing and linkage (with provision of false positive and false negative rates, comparisons of linked and unlinked data, and any sensitivity analyses).^16^ A flow diagram showing the processes for cleaning and linking different coding sources and datasets can aid understanding of the study design.
	*Reporting of the governance framework underpinning the study from a technical/data stewardship standpoint.* This includes a clear purpose for data gathering and the parameters and time limit of consent, clear mechanisms for data processing (“what happens with my data”), and a description of what the data can and cannot be used for (i.e. the mandate given for research).
2. Data security and privacy	*Working towards a new, sustainable mandate from the public and patients to use their health data may require moving away from abstract rules and regulations and towards more constructive governance, in which trust is a central concept.* The trust of patients and the public in research institutions and in science is pivotal because of the liberties they give to researchers to use their data, which are the product of a social licence based on this trust.
	*Gaining this trust would benefit from understanding what society and stakeholders expect from scientists conducting health data research, with engagement of stakeholders from the concept stage.* Co-creation of data governance based on inclusion of patient/public communities and dialogue with researchers is crucial for ethical and sustainable governance, and to translate expectations into scientific research and scientific output.
	*Researchers and big data consortia have to be mindful that trustworthiness comes with the duty to act in ethically-responsible ways.* This concerns two areas; first is the competence in data handling (meaning that systems are in place to ensure data protection and there is a framework of rules and regulations for data sharing), and second is what motivates the data analysis. Ongoing dialogue can ensure that public values continue to be aligned with the governance structures of health data research projects. Questions arise as to how to measure success at implementing public values into research, and what levels of public support are sufficient to grant a mandate for data usage.
	*Complex organisational structures may be less important for this trust than is often asserted.* Complicated rules and regulations may do more harm than good in establishing the conditions for public trust in big data health research to flourish, and as a result be counterproductive especially when a social license has not been adequately achieved.^17^
	*Embracing values such as transparency, reciprocity, inclusivity and service to the common good.* These values can be embedded into the governance framework of big data health research.^18^ This calls for constructing a narrative that researchers and research consortia can be held accountable so that patients and the wider public are willing, and consistently willing, to place their trust in health research projects.
	*Governance could be aided by developing a framework for accountability.* This includes clear distinctions between anonymised, pseudonymised and aggregate data along with plain language explanation to participants and users, and discrimination between primary and secondary use of data sources.
3. Publications using structured healthcare data	*Accountability for the source of data and how the data have been collected (traceability).* As with data security, a framework of accountability would enable editorial teams in medical journals to be aware of the technical processes prior to data analysis.
	*Sharing of data, codes and algorithms used to analyse datasets.* Similar to the requirement for pre-registration of clinical trials and pre-publication of protocols, journals could restrict publication where the coding within a study is not shared.
	*Demonstration of data validity and robust analysis.* The FDA and EMA already suggest independent checking or accreditation of data sources; this accreditation could be provided to editors to increase their confidence in data quality.
	*Balancing the speed of publication against requirements for data validation.* Prompt publication (for example of results with immediate public health implication) needs to be balanced against validation of data sources to ensure authenticity.
	*Scientific advice committees with experts in big data analytics to aid journal editorial teams.* The skill-set required in editors and reviewers for studies using structured healthcare data is not the same as having statistical or clinical trials experience; expertise in EHR data and respective coding systems could add value to the journal review process.
	*Widening gap between the knowledge of physicians and the advanced methodologies used in big data papers.* Medical/graduate students and practising clinicians, as well as hospital managers and leadership, need training in health data management and analysis. This is important to build a digital workforce with increased capacity and capability to translate publications using new approaches to improve patient care.^19^
4. Addressing the needs of regulators, reimbursement authorities and clinical practice guidelines	*EHR-based trials have the potential to generate reliable and cost-efficient results.* Each type of trial and each type of clinical question is considered in an individual context, including under what circumstances a particular type of EHR process could assist in answering questions about a particular intervention, and with what limitations.
	*Further research may help explore cases in which EHR studies produce valuable evidence, and when they might be flawed.* This will generate confidence in regulators for future EHR studies, and for guideline taskforces to appropriately appraise evidence.
	*Quality standards will help to ensure that the information recorded in EHR systems represents real events without bias.* This will enable confidence that trials using EHRs can produce reliable results on efficacy and safety, and could include examination of the validity of both data sources and data analyses.
	*Source data validation to report on appropriate computational phenotypes.* This could be supported by an independent adjudication committee to examine a subset of the EHR and confirm outcome events. The use of AI techniques could facilitate larger validation studies by automated extraction of supporting text from clinical notations. Such validation exercises can be pre-registered, for example in the form of a Study-Within-A-Trial. ^20^ Another possibility is for researchers to provide consented and anonymised gold standard cases to benchmark against, or for data from devices used to verify codes (such as lead fractures). The value of synthetic datasets for validation, which mimic real data, needs further exploration.
	*Mixed model approaches to collect data on particular endpoints.* May be valuable for situations where the EHR does not reliably collect relevant data. For example, where patients and/or clinicians are asked for information, or data are collected via wearable devices or telemonitoring. In some cases, parallel monitoring of patients alongside the EHR study may provide additional confidence (for example to identify serious unexpected adverse events). Technological advances in EHR systems will help, such as the ability to retrieve EHR data on a daily basis to support clinical trials.^21^
	*Taking advantage of the many real world data initiatives to support new research.* Government agencies, regulators, charities and professional bodies have initiated programmes for better use of real-world data that can support further activity and dissemination.

### Patient and public involvement

**Figure 2:**
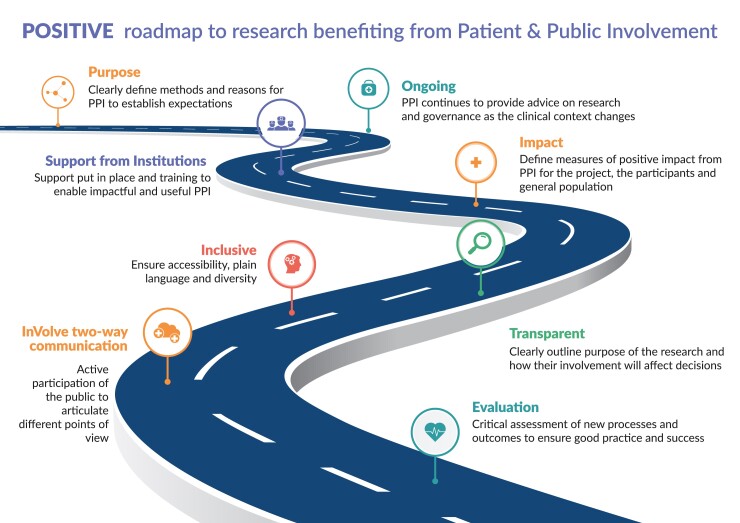
Patient and public engagement to improve clinical research. POSITIVE steps leading to co-creation with patients and the public, and better research using big data sources. Content adapted from the Consensus Statement on Public Involvement and Engagement with Data-Intensive Health Research^36^ as used in the DaRe2THINK trial programme.^27^ Adapted from Bunting et al.^37^ PPI=patient and public involvement.

The CODE-EHR consensus approach has benefited from patient and public engagement throughout the development process, including representation from the European Society of Cardiology Patient Council and the European Heart Network, an alliance of foundations and associations supporting patients and representing patient interests. We describe a potential method for engagement of the public in future research that can constructively benefit research using big data (fig 2).

## CODE-EHR reporting framework

The path from structured healthcare data to clinical research output is complex. To support further development in a transparent way, stakeholder delegates reached consensus of the need for a set of minimum standards that authors could use as a tool to enhance design, reporting, and research output. The CODE-EHR Minimum Standards Framework presented in table 2 allows authors to report on how structured healthcare data were used in their research study (either in patient identification, disease phenotyping, or outcome derivation). Preferred standards indicate high level attainment of quality and can be used as a tool to improve the future trajectory of research. The checklist was created through an iterative process based on the stakeholder proposals and covers five key areas of enhanced transparency: how and why coding was performed; the process of constructing and linking datasets; clear definitions of both diseases and outcomes; the approach to analysis, including any computational methods; and demonstrating good data governance.

**Table 2:** CODE-EHR framework: best practice checklist to report on the use of structured electronic healthcare records in clinical research

Date of completion:		Study name:		
Item	Objective	Framework standards	Minimum information to provide	Lead author acknowledgment
1. Dataset construction and linkage	To provide an understanding of how the structured healthcare data were identified and used.	Minimum: Flow diagram of datasets used in the study, and description of the processes and directionality of any linkage performed, published within the research report or supplementary documents. Preferred: Provided within a pre-published protocol or open access document.	State the source of any datasets used. Comment on how the observed and any missing data were identified and addressed, and the proportion observed for each variable. Provide data on completeness of follow-up. For linked datasets, specify how linkage was performed and the quality of linkage methods.	Choose one from: (1) Minimum standard not met (2) Minimum standard met OR (3) Preferred standard met
2. Data fit for purpose	To ensure transparency with the approach taken, with respect to coding of the structured healthcare data.	Minimum: Clear unambiguous statements on the process of coding in the methods section of the research report. Preferred: Provided within a pre-published protocol or open access document.	Confirm origin, clinical processes, and the purpose of data. Specify coding systems, clinical terminologies, or classification used and their versions, and any manipulation of the coded data. Provide detail on quality assessment for data capture. Outline potential sources of bias.	Choose one from: (1) Minimum standard not met (2) Minimum standard met OR (3) Preferred standard met
3. Disease and outcome definitions	To fully detail how conditions AND outcome events were defined, allowing other researchers to identify errors and repeat the process in other datasets.	Minimum: State what codes were used to define diseases, treatments, conditions, and outcomes *prior to statistical analysis*, including those relating to patient identification, therapy, procedures, comorbidities, and components of any composite endpoints. Preferred: Provided within a pre-published protocol or open access document *prior to statistical analysis*.	Detailed lists of codes used for each aspect of the study. Date of publication and access details for the coding manual (please add to box below). Provide definitions, implementation logic and validation of any phenotyping algorithms used. Specify any processes used to validate the coding scheme or reference to prior work.	Choose one from: (1) Minimum standard not met (2) Minimum standard met OR (3) Preferred standard met
4. Analysis	To fully detail how outcome events were analysed and allow independent assessment of the authenticity of study findings.	Minimum: Describe the process used to analyse study outcomes, including statistical methods and use of any machine learning or algorithmic approaches. Preferred: Provide a statistical analysis plan as a supplementary file, locked before analyses commencing.	Provide details on all statistical methods used. Provide links to any machine code or algorithms used in the analysis, preferably as open source. Specify the processes of testing assumptions, assessing model fit and any internal validation. Specify how generalisability of results was assessed, the replication of findings in other datasets, or any external validation.	Choose one from: (1) Minimum standard not met (2) Minimum standard met OR (3) Preferred standard met
5. Ethics and governance	To provide patients, who might or might not have given consent, and regulatory authorities the ability to interrogate the security and provenance of the data.	Minimum: Clear unambiguous statements on how the principles of Good Clinical Practice and Data Protection will be/were met, provided in the methods section of the research report. Preferred: Provided within a pre-published protocol or open access document with evidence of patient and public engagement.	State how informed consent was acquired, or governance if no patient consent. Specify how data privacy was protected in the collection and storage of data. Detail what steps were taken for patient and public involvement in the research study. Provide information on where anonymised source data or code can be obtained for verification and further research.	Choose one from: (1) Minimum standard not met (2) Minimum standard met OR (3) Preferred standard met
6. Coding manual	DOI of publication or website address: Date published:			
7. Comments				
**8. Summary declaration**	Choose one from: One or more minimum standards not met OR **All minimum standards met** Number of preferred standards met: / 5			

Directions for use:

**Research team:** To complete the checklist, authors will need to consider these points during the design of the research to ensure that coding protocols and coding manuals are pre-published. Where applicable, it is advisable that all five minimum standards are met for an individual research study, whether observational or a controlled trial. If any component is not applicable to the study, the corresponding author can indicate why this is the case in the comment box. This checklist can accompany the article as a supplementary file on submission to the journal, with the ability for readers to review responses. A comment on the meeting of standards in the text of the method section is suggested, eg; “this study meets all five of the CODE-EHR minimum framework standards for the use of structured healthcare data in clinical research, with two out of five standards meeting preferred criteria <add reference to this CODE-EHR paper; https://doi.org/10.1136/bmj-2021-069048>”; OR “this study meets four out of five of the CODE-EHR minimum framework standards for the use of structured healthcare data in clinical research; one of the five minimum standards was not met as coding schemes were not specified prior to analysis <add reference to this CODE-EHR paper; https://doi.org/10.1136/bmj-2021-069048>.” Note, easy to complete form versions of this checklist are available in appendix 4 (word version) and appendix 5 (pdf version), and also at https://www.escardio.org/bigdata.

**Research appraisers (patients, clinicians, regulators, guideline task forces):** Where applicable, it is advisable that all five minimum standards are met for the research study to be considered robust.

The framework aims to improve the quality of studies using structured healthcare data and to give confidence in their use for clinical decision making. See appendix 2 for a step-by-step approach to completion of the CODE-EHR reporting checklist, with relevant best practice examples. We also present a detailed description of the workflow that led to the checklist in appendix 3. Form versions of the checklist are provided in appendix 4 (word version) and appendix 5 (pdf version).

## Discussion

Technological progress has led to rapid evolution in heath data systems with immediate impact in daily clinical practice. The potential for improving patient care and outcomes are clear, as are the challenges and limitations to achieving this objective.^22^ Big data analytics now support large scale (and cost efficient) clinical research, with trials based within registries or the EHR itself now heralding a new era in evidence generation. These processes can be further developed by an accompanying social license and upskilling of knowledge for all stakeholders. Co-creation and shared decision making with patients and the public^23^ is an important way to ensure appropriate data stewardship and privacy, leading to clinical impact through robust publications, regulatory decision making, and practice guidelines. In this paper, we have reported on a global multistakeholder process to develop a framework for researchers to use in the design and reporting of studies that include structured or coded healthcare data.

Digital health records are confusing for most researchers, with varying access to a myriad of different coding systems and classifications, and considerable differences across (and within) countries. Linkage of different health sources is often a core component of research based on structured healthcare data, and yet, this aspect is frequently overlooked when reporting such studies. Data privacy and the license for research can be severely compromised if linkage is not secure; hence, our focus is on transparency about how data are coded and linked, and how these approaches are openly discussed and documented. The stakeholder consensus meetings highlighted this area as a key concern for future research, supported by evidence that very few studies have provided sufficient detail to understand the research process.^24,25^ The advent of registry and EHR-based randomised controlled trials^21,26,27^ reinforces the imperative to see improvements in these areas, and to define new concepts for quality research. With the development of robust analytics supported by machine learning algorithms,^28^ similar approaches have already been used to support artificial intelligence in healthcare.^29^

A lack of transparency has a direct impact on the value of research using coded records, with issues arising for medical journals, regulators, clinical guideline writers, and more generally clinicians and the public. Bringing together the full range of these stakeholders, we aimed to take full advantage of recent technical developments to use structured healthcare data for research, to approach limitations directly, and to provide a framework across all medical fields where coded data can be used to improve patient care. A number of other overlapping themes emerged from the discussions, including the generation and retainment of public trust and confidence, and the need for coherent plans to deal with data security failures. Forethought about dealing with the harmonisation of data and the requirement for embedded validation methods were highlighted as key factors for future successful research. Similarly, education and communication are crucial for patients, citizens and healthcare professionals to effectively use the results from structured healthcare data studies.

The covid-19 pandemic has illustrated the need for rapid access to routine healthcare data to guide and monitor clinical care, and a clinical trial infrastructure to allow for immediate deployment. The digitalisation of healthcare, in particular the use of EHRs, offered the clinical community a unique opportunity to develop a learning healthcare system that could efficiently address the effects of covid-19. For example, information about the relationship between covid-19 and cardiovascular disease through linked EHR data that has combined primary care data, hospital data, death records, and covid-19 testing in more than 54 million people.^30^ However, the pandemic also made clear the obstacles within various systems that restricted the sharing of data in almost real time that could direct care and help design clinical trials. Established governance, security, interoperability (system architecture that spans different EHR systems and healthcare providers), and phenotype definition, among other issues, limited access to routine EHR data especially in the first period of the pandemic.

The CODE-EHR framework is intended to complement available reporting checklists.^31–34^ Although existing checklists are aimed at transparency in the reporting of important methodological components of clinical research, the CODE-EHR framework is designed to ensure that a common set of minimum standards are applied across all research using structured healthcare data. This range includes observational studies and controlled trials, with the preferred standards giving the direction of research design for all future EHR studies. Additionally, the framework supports the wider implementation of good quality real world data research based on the FAIR data principles.^13^ Researchers are advised to use the checklist during the design phase of their study to ensure that key criteria for successful research and research impact are already embedded. The process will aid journal editors, regulators, guideline writers, clinicians, and patients to better appreciate the underpinning value, and also the limitations of the study. Dissemination plans for CODE-EHR include discussion with journals to request authors complete the checklist when submitting relevant research, attaining full registration with the EQUATOR network,^35^ and outreach via international digital health groups to engage their members and other relevant stakeholder organisations. After publication, the CODE-EHR framework will undergo a two year evaluation, including discussion with researchers using the approach, with a plan for iterative improvements to adapt to this rapidly developing field of medical research.

## Conclusion

The CODE-EHR framework was designed by a multistakeholder panel to improve design and reporting of research studies using structured electronic healthcare data. Research using these data sources is a vital component of future healthcare evaluation and delivery and will take an increasingly important role in decisions by regulatory, governmental, and healthcare agencies, as well as clinicians and patients in every medical specialty. The CODE-EHR checklist asks for clarity on reporting and defines a set of minimum and preferred standards on the processes that underpin coding, dataset construction and linkage, disease and outcome definitions, analysis, and research governance. Iterative updates to this framework are expected to enhance research quality and value and to generate new pathways for impact using routinely collected healthcare data.

## Supplementary Material

ehac426_Supplementary_Data
