# Supplementary material for: CODE-EHR best practice framework for the use of structured electronic healthcare records in clinical research
Source: Eur Heart J. 2022 Aug 29;43(37):3578–88. doi: 10.1093/eurheartj/ehac426 (PMC9452067; doi:10.1093/eurheartj/ehac426)
Supplement: ehac426_Supplementary_Data [file ehac426_supplementary_data.zip › CODE-EHR_fig1.pdf]

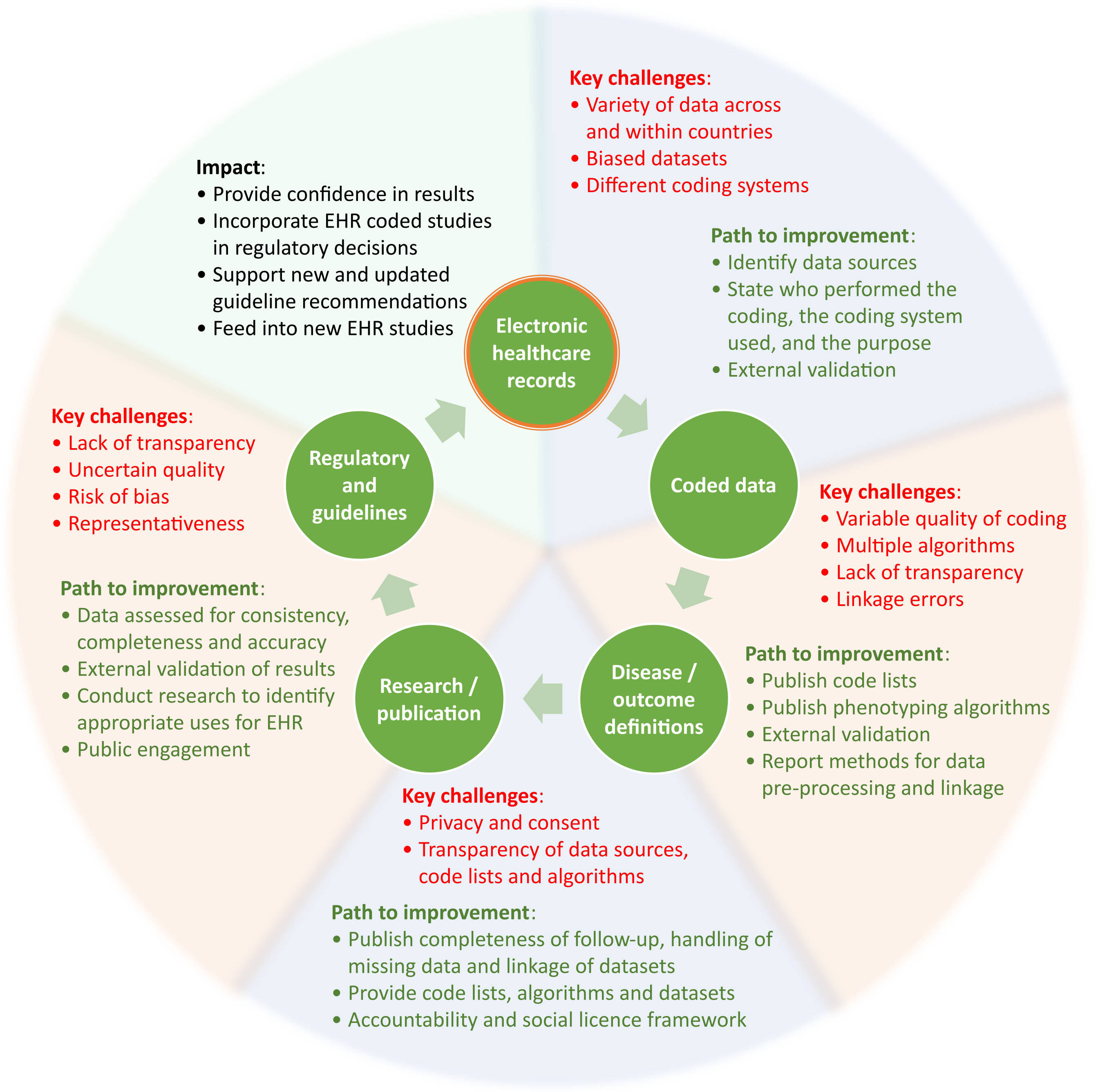
Electronic healthcare records
Key challenges:
• Variety of data across and within countries
• Biased datasets
• Different coding systems
Path to improvement:
• Identify data sources
• State who performed the coding, the coding system used, and the purpose
• External validation
Coded data
Key challenges:
• Variable quality of coding
• Multiple algorithms
• Lack of transparency
• Linkage errors
Path to improvement:
• Publish code lists
• Publish phenotyping algorithms
• External validation
• Report methods for data pre-processing and linkage
Disease / outcome definitions
Research / publication
Key challenges:
• Privacy and consent
• Transparency of data sources, code lists and algorithms
Path to improvement:
• Publish completeness of follow-up, handling of missing data and linkage of datasets
• Provide code lists, algorithms and datasets
• Accountability and social licence framework
Regulatory and guidelines
Key challenges:
• Lack of transparency
• Uncertain quality
• Risk of bias
• Representativeness
Path to improvement:
• Data assessed for consistency, completeness and accuracy
• External validation of results
• Conduct research to identify appropriate uses for EHR
• Public engagement
Impact:
• Provide confidence in results
• Incorporate EHR coded studies in regulatory decisions
• Support new and updated guideline recommendations
• Feed into new EHR studies
