# Supplementary figures and images for: CODE-EHR best practice framework for the use of structured electronic healthcare records in clinical research
Source: Eur Heart J. 2022 Aug 29;43(37):3578–88. doi: 10.1093/eurheartj/ehac426 (PMC9452067; doi:10.1093/eurheartj/ehac426)

# POSITIVE roadmap to research benefiting from Patient & Public Involvement

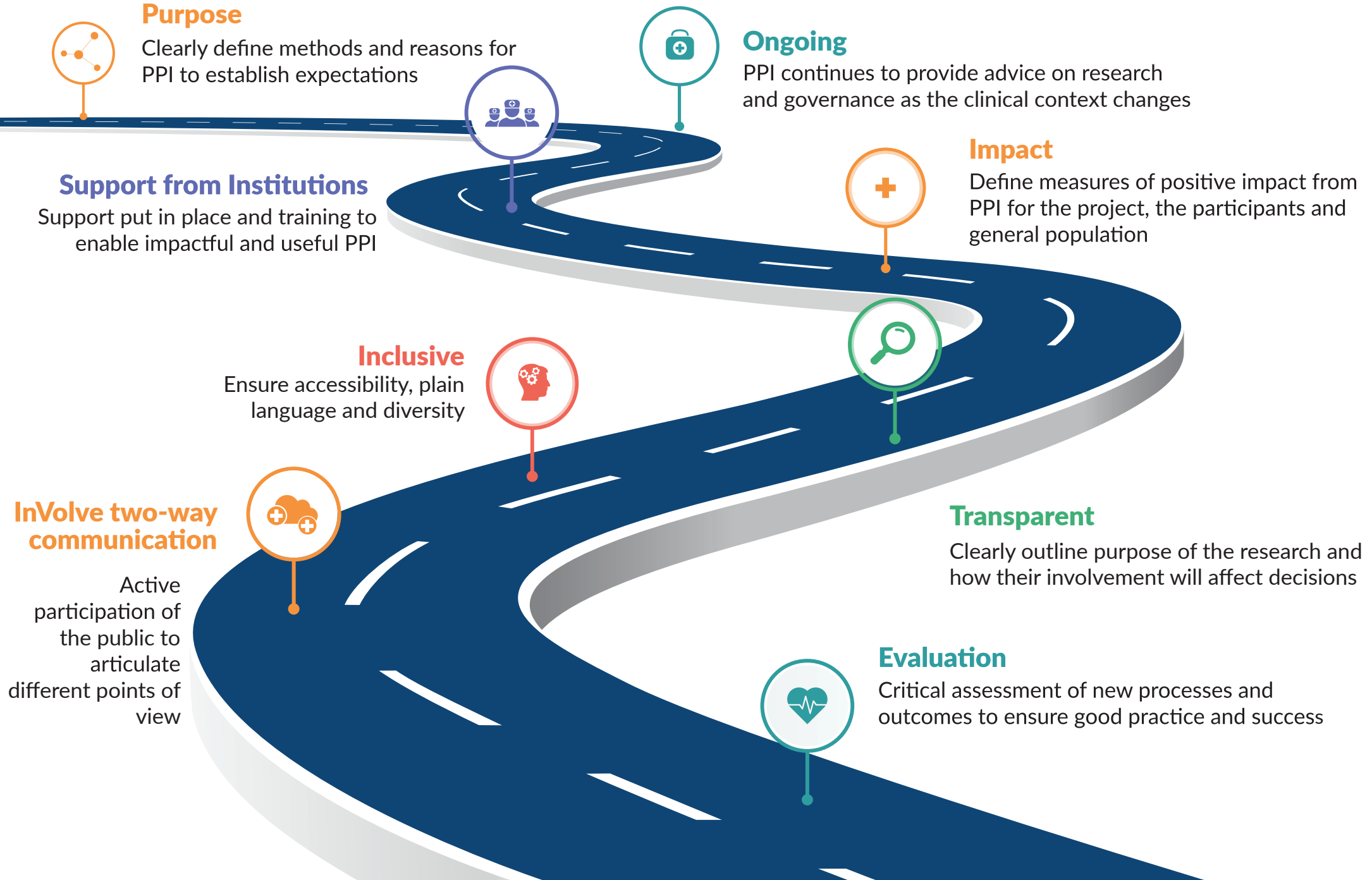

Supplement: ehac426_Supplementary_Data [file ehac426_supplementary_data.zip › CODE-EHR_fig2.pdf]
